# Clinical application of risk assessment in PAH: Expert center APRN recommendations

**DOI:** 10.1002/pul2.12106

**Published:** 2022-07-01

**Authors:** Melisa Wilson, Jennifer Keeley, Martha Kingman, Susanne McDevitt, Jacqueline Brewer, Frances Rogers, Wendy Hill, Zachary Rideman, Meredith Broderick

**Affiliations:** ^1^ AdventHealth Orlando Florida USA; ^2^ Allegheny Health Network, Allegheny General Hospital Pittsburgh Pennsylvania USA; ^3^ University of Texas Southwestern Medical Center Dallas Texas USA; ^4^ Michigan Medicine Ann Arbor Michigan USA; ^5^ Beaumont Health Troy Michigan USA; ^6^ Temple University Hospital Pulmonary Hypertension, Right Heart Failure and CTEPH Program Philadelphia Pennsylvania USA; ^7^ Cedars Sinai Medical Group Los Angeles California USA; ^8^ United Therapeutics Corporation Silver Spring Maryland USA

**Keywords:** mortality risk assessment, multiparameter risk assessment, pulmonary arterial hypertension, REVEAL, risk assessment tools

## Abstract

Performing longitudinal and consistent risk assessments for patients with pulmonary arterial hypertension (PAH) is important to help guide treatment decisions to achieve early on and maintain a low‐risk status and improve patient morbidity and mortality. Clinical gestalt or expert perception alone may over or underestimate a patient's risk status. Indeed, regular and continued use of validated risk assessment tools more accurately predict patients' survival. Effective PAH risk assessments are often underutilized even though many seasoned clinicians will attest to using these tools routinely. We present recommendations based on real‐world experience in varied clinical practice settings around the United States for overcoming barriers to facilitate regular, serial formal risk assessment. Expert advanced practice provider clinicians from mid to large‐size medical centers collaborated to formulate recommendations based on multiple discourses and discussions. Enlisting the help of support staff, such as medical assistants and nurses, to fill in available risk parameters in risk assessment tools can save time for providers and increase efficiency, as can technology‐based solutions such as integrating risk assessments into electronic medical records. Modified, abbreviated risk assessment tools can be applied to a patient's clinical scenario when all of a patient's data are not available to complete a more comprehensive assessment. Initial discussions regarding the overall meaning and prognostic importance of risk scores may assist patients to take on a more active role in terms of informed decision‐making regarding their care. A collaborative approach can help clinics establish consistent use of risk assessment.

## INTRODUCTION

Pulmonary arterial hypertension (PAH) is a progressive, debilitating, and often fatal disease characterized by narrowing of the pulmonary vasculature, leading to increased pulmonary arterial pressure, pulmonary vascular resistance, and ultimately right heart failure and death.^1^, ^2^ Several studies have found that patients with mild symptoms can have a significant underlying disease, increasing their mortality risk,^3^, ^4^, ^5^, ^6^, ^7^, ^8^, ^9^ suggesting that pulmonary vascular dysfunction precedes clinical signs of PAH.^10^ Thus, early and accurate use of formal risk assessment tools is vital to discern early disease, monitor for disease progression, guide treatment decisions, and assess whether treatment goals are being met. Patients who are deemed “low risk” demonstrate a lower mortality rate, increased exercise capacity, and better quality of life.^11^, ^12^ With certainty, the use of risk assessment tools enhances a clinicians' ability to evaluate patient response to treatment and improve timely identification of those patients who may be slowly decompensating.^11^

The 2015 European Society of Cardiology/European Respiratory Society Pulmonary Hypertension guidelines, along with other expert panels, recommend that comprehensive assessments should be conducted every 3–6 months in stable patients, and after right heart catheterization, using multiple parameters, including invasive and noninvasive variables such as biomarkers, clinical status, exercise testing, echocardiography, and hemodynamic evaluation.^11^, ^13^, ^14^, ^15^, ^16^, ^17^, ^18^, ^19^, ^20^ No single variable is sufficient to provide prognostic information.^11^

Studies have shown that clinical gestalt alone is accurate in only 45% of assessments and can under or overestimate a patient's risk.^2^ Consistent evaluation of patients' risk assessment by utilizing comprehensive or abbreviated risk assessment tools can help clinicians reliably identify patients at higher risk that may have been misclassified using other methods.^21^ Over the past decade, several risk assessment tools have been created using patient data from large patient registries to identify objective metrics to assess disease prognosis. See Table 1 for a list of risk assessment tools.

**Table 1:** Risk assessment tools

		Variables														
Tool	Length	WHO group 1 subgroup	Demographics and characteristics	NYHA/WHO functional class	Vital signs (systolic BP, heart rate)	6MWD	BNP/NT‐proBNP	Echocardiogram	Pulmonary functional test	Renal insufficiency	PVR*	RAP*	Cardiac index*	S_v_O_2_ *	All‐cause hospitalization ≤6 months	
REVEAL^9^	Full	X	X	X	X	X	X	X	X	X	X	X				
REVEAL 2.0^22^	Full	X	X	X	X	X	X	X	X	X	X	X			X	
SPAHR^23^	Full			X		X	X	X				X	X	X		
French Invasive^24^	Full			X		X						X	X			
REVEAL Lite 2^25^	Mod			X	X	X	X			X						
COMPERA^26^	Mod			X		X	X					X	X	X		
French noninvasive^24^	Mod			X		X	X									

Abbreviations: 6MWD, 6‐minute walking distance; BNP,  B‐type natriuretic peptide; BP, blood pressure; Mod, modified; NT‐proBNP, N‐terminal (NT)‐prohormone BNP; NYHA, New York Heart Association; RA, right atrial; RAP, right atrial pressure; REVEAL, Registry to Evaluate Early And Long‐term PAH Disease Management; S_v_O_2_, mixed venous oxygen saturation; WHO, World Health Organization.

* Right heart catheterization needed to obtain this information.

Care centers can use risk assessment to help standardize care and as a guiding tool to establish the need to escalate therapy, refer to transplant, and have advanced care planning discussions. In a clinical PAH care setting, a multiparametric risk assessment should take place at the first clinical visit, all subsequent visits, and should be performed more frequently for patients who demonstrate signs of clinical worsening. However, numerous barriers to the consistent use of risk assessment tools have been identified by PAH providers, including time constraints, lack of administrative support, complexity of scoring systems, insufficient awareness and training, lack of integration with existing technology systems, and an absence of clarity on which tool to use.^21^

A recent survey administered to 121 PAH providers in the United States aimed at describing the current clinical utilization of risk assessment tools in PAH and found that only 59% of treatment decision‐makers reported using formal tools to assess risk in their PAH patients.^21^ The rate of tool use was lower for nonphysicians (48%) than for physicians (65%) and slightly lower for treatment decision‐makers with <5 years of experience treating PAH.^21^ The rate of risk assessment tool used was also surprisingly lower at PAH accredited centers (52%) than at nonaccredited centers (66%), particularly among physicians.^21^ Risk was most frequently assessed by providers at the time of diagnosis (54%) and at the time of worsening symptoms, with only 19% of clinics reporting regular use, implying low use of risk assessment tools and lack of consistent use in clinical practice.^21^, ^27^

**Figure 1:**
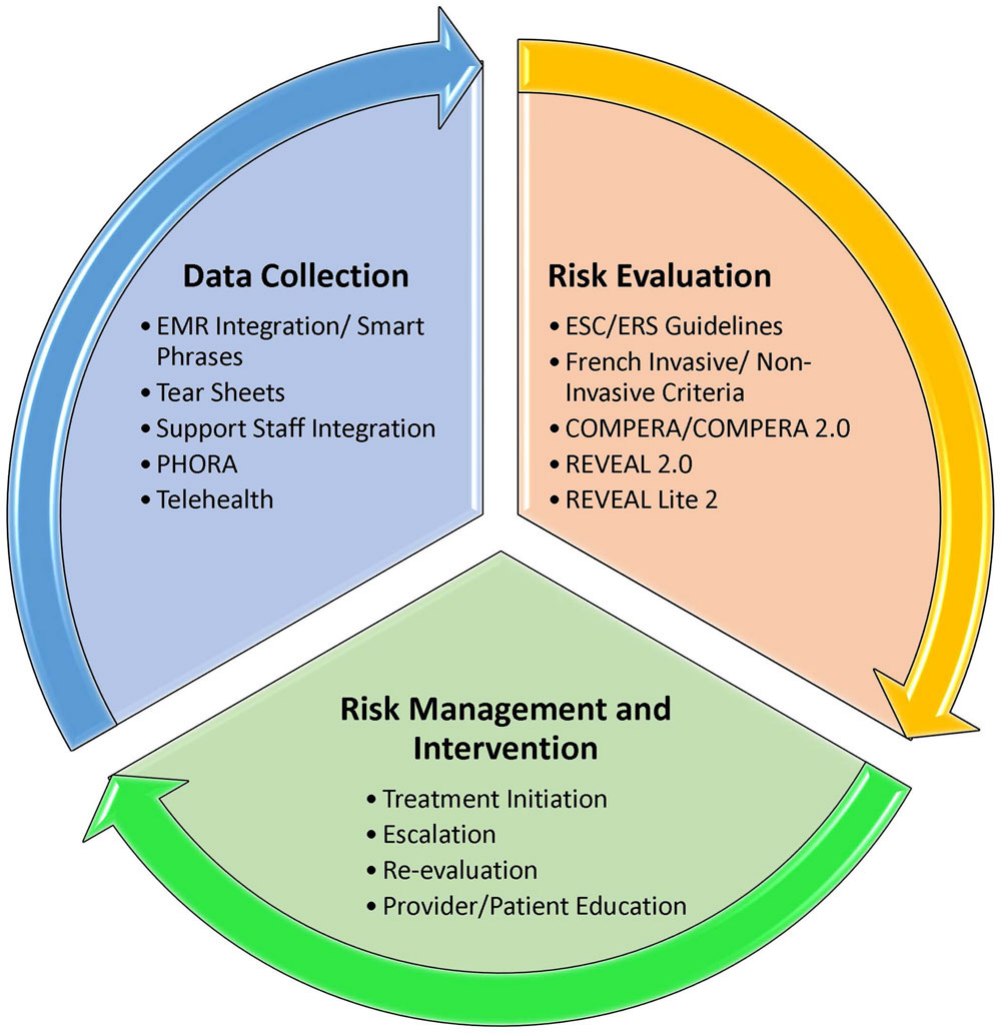
Risk assessment framework. EMR, electronic medical records; ERS, European Respiratory Society; ESC, European Society of Cardiology; PHORA, Pulmonary Hypertension Outcomes Risk Assessment

The aim of this publication is to share expert clinician recommendations based on real‐world experience to overcome barriers to incorporating risk assessment into clinical practice, to improve patient outcomes. A cohort of seven APPs provided recommendations for implementation of risk assessment. These clinicians represented varying healthcare settings from different geographies and were selected based on their experience implementing formal risk assessment and expertise in the management of patients with PAH. Recommendations were formulated based on multiple discussions (Figure 1).

## LACK OF TIME PREVENTS THE REGULAR USE OF RISK ASSESSMENTS

### Recommendation: Technology‐based solutions as a time‐saving tool

Lack of time can be a challenge when implementing change in a clinical setting. Previous research indicates time constraints as the most cited barrier to implementation of risk assessment.^21^ It is estimated to take approximately 2 min to extract the necessary variables from patient charts into the online PHORA calculator to evaluate risk. Utilizing technology‐based solutions can help improve time management and feasibility of incorporating risk assessment into routine clinical practice. Computer applications, such as electronic alerts and reminders when predetermined risk assessments are expected, as well as the integration of PAH risk assessment tools into electronic medical records (EMRs), are ways to overcome these barriers.

Risk assessment tools can be incorporated into EMR systems by using predetermined text (often termed smart or dot phrases) in clinic notes and calculating risk scores based on manually entered variables. EMR technical support departments can provide guidance on how to deploy and optimize these phrases to meet individual clinics needs and create a path that will input requested data. Publicly accessible tools are available to create pursuits in Epic and Cerner, two types of EMR systems utilized by medical facilities, to identify patients based on their risk status variables.^28^

Smart or dot phrases (shortcuts to document frequently used phrases or information in the EMR) also provides a method for standardized documentation, also lending opportunities for patient safety and quality improvement initiatives. Importantly, they provide a method for quick, efficient, and reproducible documentation including point of care and evidenced‐based decision‐making and prognostic tools.

It is crucial to clearly identify even small incremental changes in risk scores longitudinally to determine patient prognosis, monitor disease progression, and make prompt validated treatment decisions. Clinics who do not yet use an EMR system or are unable to set up smart or dot phrases may choose to employ web‐based calculators as an alternative technology solution. The Pulmonary Hypertension Outcomes Risk Assessment web portal, a currently available online resource with ongoing development, allows clinicians to enter patient variables and automatically determine their risk score using a variety of risk assessment tools.^29^ Other websites also have automated risk assessment calculators available for a variety of tools, downloadable tear pads, and risk calculation sheets (see Appendix A and B).^28^

### Recommendation: Integrating support staff

Enlisting the help of support staff, such as medical assistants or nurses, to assist with adding test results into a risk assessment tool before the patient being seen by the provider increases efficiency with routine risk stratification. As a time‐saving measure, support staff can review charts before a visit to provide the necessary variables to prepopulate a risk calculator. In this scenario, the provider may only need to add the NYHA/WHO functional class parameter to calculate the risk score. For clinics using EMRs that do not have this capability, support staff may need to mine the data to find relevant results and ensure they are documented in the chart before the patient visit.

## INSUFFICIENT AWARENESS AND TRAINING

### Recommendation: Provider education

While there are a growing number of learning opportunities related to increasing the understanding of risk assessment in PAH, there may be a lack of awareness about available resources to integrate use in clinical practice. Discussion of how and when to use these resources may increase use of risk assessment tools. Increasing provider education and mitigating common knowledge barriers may help advance understanding and implementation.

Risk assessments may be completed and scored by an advanced practice provider (APP), physician, or other provider who can determine functional class. Equipping physicians and APPs with the knowledge and resources needed to perform risk assessments can help save time, encourages the routine/consistent use of such tools, and promotes evidence‐based care for PAH patients.

Detailed, specific, and reproducible documentation of variables to calculate risk scores can also help with continuity and tracking, especially in clinics that use multiple risk assessment tools if all variables are not available at the time of the clinic appointment. In these cases, disclosing the variables used and noting any missing information can assist clinicians performing future risk assessments as they will have a better understanding of how past scores were determined.

### Recommendation: Patient education

It is important to communicate directly with patients regarding risk assessment outcomes.^30^, ^31^ Results should be communicated at a level patients can understand, using compassionate and patient‐centered communication principles. Given the fact that patients seek medical care at expert PH centers with the hope of advanced treatment options, the clinician should balance the science and the evidence of high‐risk stratification patient outcomes as “probable” with the hope of the “possible.”^32^ Informing patients that their disease is high‐risk at index evaluation or later in the disease trajectory should be done with skilled communication and empathy.

Just as some patients choose to track their 6MWD and pulmonary artery pressure, patients with adequate health literacy and support systems may also follow their risk score. Discussing risk with patients may encourage engagement and active participation in their care management. This may give healthcare providers an earlier opportunity to discuss potential prognosis and involve patients in treatment decisions.^33^ An informed patient may be more likely to advocate and for and take an active part in their care.^34^, ^35^, ^36^, ^37^

Patient understanding of disease prognosis is important to optimize informed treatment decisions. A “teach‐back” method can help providers assess if their patient has understood the conversation. Open‐ended questions in assessing patient understanding are also helpful in assessing patient understanding. The healthcare provider should also attempt to use creative but appropriate health literate measures to demystify complex health conversations. Providers should strive to be culturally competent to help all patients understand risk score outcomes and make informed decisions regarding their disease and overall health. The use of illustrations, pictures, or electronic devices can be helpful options for educating patients.

### Recommendation: Increasing provider autonomy

In clinics that do not routinely use a specific risk assessment tool or lack consensus on patterns of use, providers may feel ambiguous regarding when and how to perform a risk assessment. However, a collaborative approach that promotes provider autonomy and the inclusion of APPs clinical judgment may promote an environment to increase the use of risk assessment tools.

A study of a newly established APP clinic for PAH follow‐up care found that by increasing APPs' autonomy, patient outcomes were improved.^38^ By utilizing a full‐provider team and completing a risk assessment at every visit, 1‐year survival was increased.^38^

### Recommendation: Telehealth

Given the SARS‐CoV‐2 pandemic, most institutions have made changes to accommodate patient care from a distance. Pulmonary hypertension providers began conducting follow‐up visits virtually, without repeat testing, necessitating the use of an abbreviated risk assessment.

Increased accessibility to personal devices, such as smartphones and smartwatches/bands, step counters, and Bluetooth‐capable ambulatory devices also provides access to a wealth of information that may be complementary in clinical settings. Many of these devices capture the biometric data needed for risk assessments.^39^, ^40^ Consumer‐based accelerometers for 6MWD,^41^ recently validated patient‐assessed functional class questionnaires,^42^ blood pressure cuffs,^43^, ^44^ and satellite laboratory companies for assessing B‐type natriuretic peptide or N‐terminal prohormone brain natriuretic peptide can also be incorporated to facilitate telehealth care. Conversely, the lack of technological devices remains a limitation for a large percentage of the population, adding to the digital access divide and this limitation must be considered in PH program risk assessment.

There has been increased clinician interest, outside of the traditional American Thoracic Society (ATS) medically supervised 6‐min hall walk test to evaluate exercise and functional capacity. A recent study found that a mobile‐based 6MWD is feasible and accurate in a home‐based environment.^45^ This mobile application has not been validated for patients with PAH Self or family/caregiver‐directed functional tests such as the incremental shuttle walk test, offer a low‐tech option for an objective functional assessment when a face‐to‐face 6MWD is not feasible.^46^ This approach may further impact the PH clinician's ability to perform serial risk assessment during the era of expanding telehealth care.

## SUMMARY

Patients with baseline or achievable low‐risk stratification have improved survival outcomes. Conducting routine risk assessment provides clinicians the ability to objectively assess PAH patients' mortality risk and may guide treatment decisions.^27^, ^47^ Therefore, it is of paramount importance to conduct regular risk evaluations at least every 3–6 months on patients with PAH to identify and mitigate early disease progression. Of course, more frequent assessments may be needed if PAH therapy is escalated, or disease worsening is identified. Consistently incorporating a risk assessment tool as part of clinical visits, similar to a physical exam or taking a history, may enable clinicians to make timely, informed treatment decisions that ultimately impact outcomes.

Barriers to risk assessment vary depending on geographic location including access to clinical tests, trained clinical personnel, and digital tools needed to assess risk. However, different risk calculators utilize different clinical variables, making it possible to perform risk evaluation based on data availability. While there are multiple barriers to consistent risk assessment, strategies to increase access, feasibility, and efficiency, of validated risk assessment tools should be implemented. Technology‐based solutions such as EMR integration and enlisting the help of nurses and administrative staff can help improve time management.

Efficiently utilizing a risk assessment tool, including obtaining a baseline index assessment and regular, serial follow‐up assessments, will help patients achieve and maintain a low‐risk status, and increase progression‐free survival intervals.^8^, ^11^, ^27^, ^38^, ^48^, ^49^, ^50^ Patient motivation and compliance may also be improved by increasing patient engagement with regard to their risk status and therapy goals.^33^

Consistent and longitudinal use of risk assessment tools will help to identify the need to escalate therapy, facilitate important therapy conversations, and ultimately may support more timely movement toward low‐risk status which portends improved prognosis. Establishing a “new normal” in documentation will become second nature similar to documenting the WHO (World Health Organization) group and WHO functional class. Slowing the progression of PAH and impacting patients' ability to achieve low‐risk status is the ultimate goal as we strive to transform PAH into a chronic manageable, life‐long disease.

## AUTHOR CONTRIBUTIONS

Melisa Wilson, Jennifer Keeley, Martha Kingman, Susanne McDevitt, Jacqueline Brewer, Frances Rogers, and Wendy Hill made substantial contributions to the conception and design of the work. Melisa Wilson, Jennifer Keeley, Martha Kingman, Susanne McDevitt, Jacqueline Brewer, Frances Rogers, Wendy Hill, Zachary Rideman, and Meredith Broderick contributed to the drafting and revising of the manuscript.

## CONFLICTS OF INTEREST

Melisa Wilson is a speaker and consultant for United Therapeutics and Bayer Pharmaceuticals. Melisa Wilson has been an advisor for Janssen. Martha Kingman has been a past advisor to United Therapeutics Corporation, Actelion/Janssen, and Bayer. Susanne McDevitt is a scientific advisor to Acceleron Pharma, Bayer, Janssen Pharmaceuticals, and United Therapeutics Corporation. Jacqueline Brewer is a speaker and consultant for United Therapeutics Corporation. Frances Rogers is a speaker for United Therapeutics Corporation. Zachary Rideman and Meredith Broderick are current employees at United Therapeutics Corporation. Jennifer Keeley is a speaker for Janssen Pharmaceuticals, Bayer Pharmaceuticals, and is an advisor to Janssen Pharmaceuticals.

## ETHICS STATEMENT

Ethics review and approval was not required for this expert opinon.
